# Self-Referenced and Wide-Range Tunable Microwave Frequency Measurement Using Period-One Oscillation and Spectral Gating

**DOI:** 10.3390/s26113403

**Published:** 2026-05-27

**Authors:** Zhangyi Yang, Zuoheng Liu, Wei Dong

**Affiliations:** State Key Laboratory of Integrated Optoelectronics, JLU Region, College of Electronic Science and Engineering, Jilin University, Changchun 130012, China

**Keywords:** frequency measurement, frequency-to-time mapping, microwave photonics, optical injection, period-one oscillation

## Abstract

**Highlights:**

**What are the main findings?**

- Proposed an all-optical, self-referenced microwave frequency measurement scheme utilizing laser period-one dynamics combined with spectral gating mechanism.
- Achieved a flexibly reconfigurable measurement range of 10~48 GHz, delivering frequency resolution of 50 MHz and root-mean-square error of less than 15 MHz.

**What are the implications of the main findings?**

- Eliminated reliance on high-speed electrical sources and external synchronization by directly extracting negative pulses as temporal markers for optical injection cycle.
- Enabled time–frequency analysis of multi-tone and complex transient signals, offering a broadband FTTM solution for radar and electronic warfare.

**Abstract:**

We demonstrate a reconfigurable microwave frequency measurement (MFM) scheme based on the period-one (P1) dynamics of an optically injected semiconductor laser. Unlike conventional architectures relying on electrical frequency-sweeping, our approach utilizes the P1 oscillation to generate a wideband linear optical chirp. A spectral gating mechanism is introduced, where an optical bandpass filter creates a negative temporal marker by rejecting free-running component of distributed feedback laser (DFB), thereby eliminating the need for external synchronization or pilot tones. The measurement range is flexibly tunable by adjusting the injection parameters, enabling a measurement range from 10 to 48 GHz. Experimental results demonstrate a frequency resolution of 50 MHz with chirp rate of 1 GHz/μs and a root-mean-square (RMS) error below 15 MHz, confirming the validity of this all-optical, self-referenced frequency-to-time mapping technique.

## 1. Introduction

Wideband microwave frequency measurement (MFM) has become increasingly important in modern radar, electronic warfare, and spectrum surveillance systems, where broadband, multi-tone, and rapidly varying signals should be identified in real time [1,2]. Microwave photonic approaches are well suited to this task because they offer broad bandwidth, low loss, and strong immunity to electromagnetic interference [3,4]. In essence, photonic MFM maps unknown microwave frequency onto another physical quantity that is easier to detect. From this perspective, reported schemes can generally be classified as frequency-to-power mapping (FTPM) [5,6], frequency-to-space mapping (FTSM) [7,8], and frequency-to-time mapping (FTTM) [9,10,11,12,13,14,15]. FTPM schemes use frequency-dependent transmission, gain, phase, or polarization responses to translate microwave frequency into optical or electrical power variations [5,6]. These schemes can achieve high measurement accuracy for single-tone signals, particularly when narrowband optical effects or steep filter slopes are used. However, their monotonic measurement range is often limited, and simultaneous multi-tone identification is difficult because different frequency components may be projected onto the same scalar response. By contrast, FTSM and channelized schemes distribute different frequency components into different wavelength or spatial channels, which is advantageous for instantaneous spectral discrimination [7,8]. Nevertheless, they usually require carefully designed multi-channel devices and additional calibration, and the trade-off among channel number, resolution, and implementation complexity remains significant.

Among these approaches, FTTM is particularly attractive because it converts microwave frequency information into temporal positions that can be read out using low-speed detection, thereby naturally supporting multi-tone measurement and time–frequency analysis of dynamic signals. Existing FTTM methods can be further divided into dispersive Fourier transform schemes [9] and frequency-sweeping filtering schemes [10,11,12,13,14,15]. In dispersive Fourier transform schemes, microwave spectral information is projected into the temporal domain through dispersive or equivalent optical processing. Such schemes are attractive for real-time time–frequency processing. However, they generally rely on carefully designed dispersive media and calibration. As a result, their achievable resolution and measurement accuracy are constrained by dispersion engineering, temporal broadening, and accumulated mapping errors. By contrast, frequency-sweeping filtering schemes perform FTTM by sweeping a frequency-selective response across the signal spectrum and identifying the temporal position of the corresponding output pulse. Owing to their relatively straightforward implementation, pulse-based readout, and flexibly configurable measurement window, these schemes have attracted particular attention. However, conventional frequency-sweeping FTTM architectures still face two major challenges: a limited measurement range and the need for a reliable time reference.

The range limitation mainly originates from the sweep-generation stage. In many reported implementations, the chirped waveform used for FTTM is generated electrically, so the accessible measurement range is ultimately constrained by the bandwidth of high-speed arbitrary waveform generators and associated RF components. To break this electronic bandwidth barrier, photonic sweep generation has been investigated [13,14,15]. Directly modulated lasers [13] can generate self-referenced sweeps but are limited to a narrow analysis bandwidth about 10 GHz by relaxation oscillation of the laser. Conversely, period-one (P1) dynamics of optically injected lasers can produce ultra-wideband chirps. However, existing P1-based schemes have not fully addressed the synchronization and flexibility challenges. The approach in [14] relies on external instrument triggers and is confined to a fixed measurement window dictated by a static phase-shifted fiber Bragg grating (PS-FBG), failing to exploit the flexible tunability of P1 dynamics. Similarly, while [15] extends the bandwidth, it requires complex multi-pulse joint analysis and external synchronization, compromising real-time performance.

The temporal reference problem arises because accurate frequency retrieval requires a stable time marker for each sweep period. The mainstream approaches to addressing this challenge include bidirectional frequency sweeping, external triggering, and pilot tone injection. Bidirectional sweeping reduces frequency ambiguity at the cost of duty cycle and suffers from pairing errors in multi-tone scenarios. External triggers provide a reliable reference but add hardware complexity. Pilot tone injection enables flexible self-referencing, but it typically requires a dedicated RF pilot source. To address this issue, our previous work [10] employed the optical carrier as the pilot tone, thereby eliminating the need for an additional RF source. Our subsequent work [12] realized self-referenced FTTM using amplitude-modulated linear frequency modulation (AM-LFM). In that scheme, a zero-voltage gap was embedded in the driving waveform to generate a negative reference pulse and eliminate external synchronization. Nevertheless, both approaches still relied on electrically generated high-speed sweeps, so the accessible measurement range remained fundamentally limited by the bandwidth of the electrical sweep-generation chain. Consequently, no reported approach has simultaneously resolved both the range limitation and the temporal reference problem.

In this article, we propose a self-referenced and wide-range tunable MFM scheme by combining P1-dynamics-based optical chirp generation with spectral gating in an FTTM architecture. The frequency sweep is provided by an optically generated chirp derived from the P1 dynamics of an injected DFB laser, thereby avoiding direct high-speed electrical chirp synthesis. In addition, a spectral gating mechanism converts the P1 injection cycle into a negative temporal marker, enabling self-referenced operation without external triggers or auxiliary pilot tones. By tuning the injection parameters, the measurement range can be flexibly reconfigured, providing a scalable solution for wideband microwave frequency analysis.

The principle of the proposed scheme will be described in detail in Section 2. The details of experimental results will be presented and discussed in Section 3. Section 4 concludes this paper.

## 2. Principle of Operation

**Figure 1:**
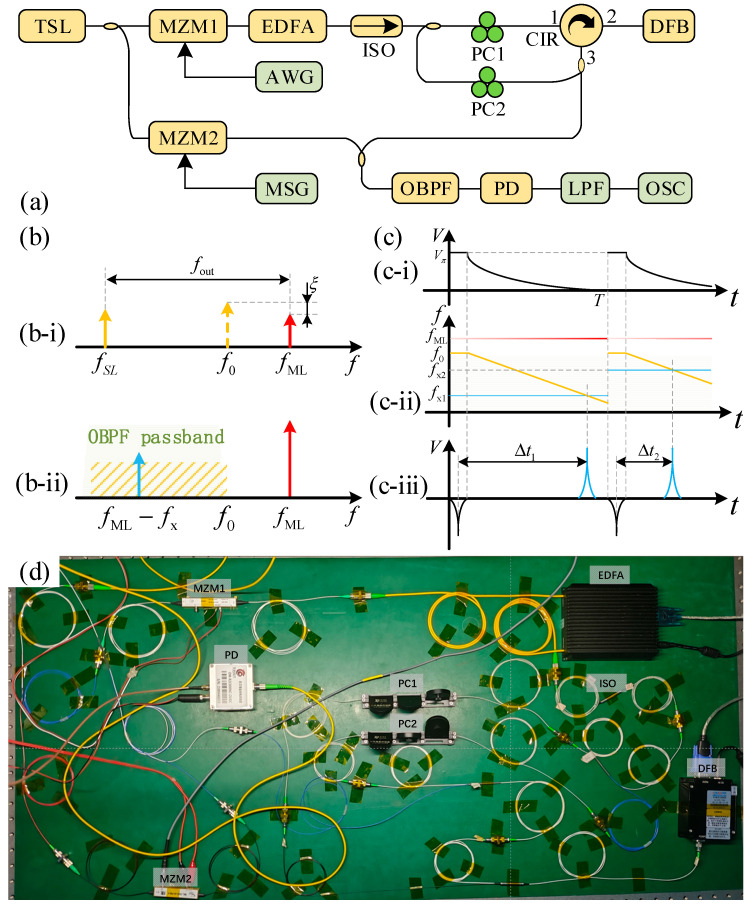
Architecture and operating principle of the proposed scheme. (**a**) Schematic of the proposed system. (**b**) Optical spectra for light (**b-i**) from port 3 of the circulator and (**b-ii**) after the OBPF. (**c**) Operating principle: (**c-i**) driving waveform of MZM1, (**c-ii**) instantaneous frequency of key signals, (**c-iii**) waveform from PD. TSL: tunable semiconductor laser, MZM: Mach–Zehnder modulator, EDFA: erbium-doped fiber amplifier, DFB: distributed feedback laser, ISO: isolator, PC: polarization controller, CIR: circulator, OBPF: optical bandpass filter, PD: photodetector, AWG: arbitrary waveform generator, MSG: microwave signal generator, OSC: oscilloscope. (**d**) Photo of experimental setup.

The schematic of the proposed system is illustrated in Figure 1a. A tunable laser (TSL-550, Santec, Komaki, Japan) acts as the master laser (ML). In the upper branch, MZM1 (AM20, AFR, Hong Kong SAR, China) is driven by a 2.5-GSa/s AWG (81160A, Keysight, Santa Rosa, CA, USA) to modulate the optical intensity. The modulated light is then amplified by an EDFA (KG-EDFA-B, Conquer, Beijing, China) and injected into the slave DFB laser (SL, KG-DFB-1550, Conquer, Beijing, China) to control the injection strength. A portion of the DFB output is tapped from the third port of the circulator and reinjected via an optical feedback loop, narrowing the instantaneous linewidth of the generated sweeping sideband effectively [16]. PC1 and PC2 are used to align the polarization states of the injected light and the feedback light with the dominant lasing polarization mode of the DFB laser, respectively, thereby improving the effective injection and feedback coupling efficiencies. In the lower branch, the signal under test (SUT) from MSG is modulated onto the optical carrier using MZM2 (IML-1550-40-PM-V, Optilab, Phoenix, AZ, USA) biased at the quadrature point. The signals from both branches are combined and processed by an OBPF (BVF-300CL, Alnair, Minato, Japan). The output is then detected by a photodetector (OM-R2C000NC, Tianqi, Zhuhai, China) and captured by an oscilloscope (MSOV254A, Keysight, Santa Rosa, CA, USA) after a 30 MHz lowpass filter. Therefore, the upper branch generates the P1-induced sweeping optical sideband, while the lower branch generates the SUT sideband. The OBPF acts as a spectral gate that selects these two sidebands for beating detection and rejects undesired components. A photo of the experimental setup is shown in Figure 1d.

The spectral characteristics of the DFB in P1 oscillation state are presented in Figure 1(b-i). Under optical injection of ML at $f_{ML}$, the DFB cavity resonance is red-shifted from its free-running frequency $f_{0}$ toward $f_{SL}$ due to the carrier-induced refractive index change, producing spectral components symmetrically located around $f_{ML}$ with unequal intensity. The frequency spacing corresponds to the P1 oscillation frequency $f_{o}=f_{ML}-f_{SL}$. Since $f_{o}$ depends on both injection strength ξ and detuning frequency $f_{i}=\left|f_{ML}-f_{0}\right|,$ the P1 oscillation frequency can be widely tuned by adjusting the injection conditions, enabling optical generation of wideband reconfigurable frequency-sweeping sideband. By applying a pre-distorted waveform to MZM1, the injection power is modulated to control $f_{SL}$, generating a linear frequency sweep across the orange area in Figure 1(b-ii). Meanwhile, the SUT at frequency $f_{x}$ modulates the ML carrier via MZM2, producing a lower first-order sideband at $f_{ML}-f_{x}$ indicated by the blue arrow. The OBPF passband represented by the green area in Figure 1(b-ii) is tuned to transmit both the P1-induced sweeping sideband and SUT lower sideband while suppressing other spurious spectral components. This configuration enables the self-referenced measurement process illustrated in Figure 1c. As shown in Figure 1(c-i), the AWG driving waveform is designed with a brief interval at $V_{\pi }$ within each period T, during which the MZM1 transmission is minimized and the optical injection into the DFB is effectively suppressed. In the absence of injection, the frequency of DFB reverts to its free-running state at $f_{0}$, which falls outside the OBPF passband as shown in Figure 1(b-ii). Consequently, optical power transmitted to the PD through the OBPF drops sharply, producing the negative reference pulses. During the remaining portion of each period, the pre-distorted MZM1 driving waveform ensures a linear frequency sweep of the generated sweeping sideband, as illustrated by the orange trace in Figure 1(c-ii). When the instantaneous frequency of the sweeping sideband approaches that of the SUT lower sideband, their beating produces positive pulses at the PD output shown in Figure 1(c-iii). For instance, two SUTs at frequencies $f_{x1}$ and $f_{x2}$ yield blue positive pulses at distinct temporal positions, with time delays $\Delta t_{1}$ and $\Delta t_{2}$ relative to the black negative reference pulse, respectively. Taking the negative pulse as the temporal reference, the instantaneous P1 oscillation frequency can be expressed as $f_{o}(t)=f_{i}+kt$. When the sweeping sideband $f_{ML}-f_{o}(t)$ overlaps with the SUT lower sideband $f_{ML}-f_{x}$, a positive beating pulse is generated at the PD output. Therefore, the SUT frequency is determined by:

(1) $$f_{x}=f_{i}+k\cdot \Delta t$$

where k is the chirp rate of the sweeping sideband, and Δt is the time delay between the negative reference pulse and the positive beating pulse.

## 3. Experimental Results

**Figure 2:**
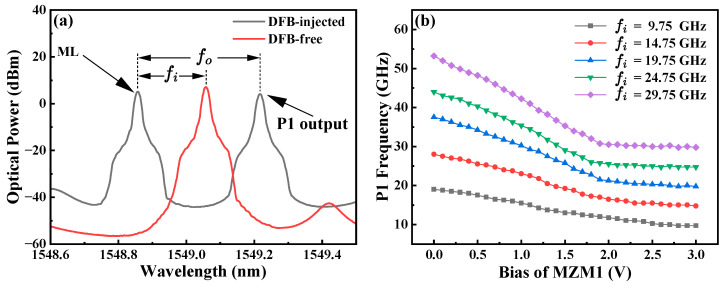
Optical injection characterization. (**a**) Spectrum of the DFB in free-running (red) and P1 oscillation (black) states. (**b**) P1 frequency versus MZM1 bias voltage for $f_{i}$ = 9.75, 14.75, 19.75, 24.75, and 29.75 GHz.

To characterize the P1-induced sweeping sideband, the optical spectra of DFB in free-running and P1 oscillation states are measured using an optical spectrum analyzer, as shown in Figure 2a. A pronounced red shift in the DFB output is observed under optical injection. To determine the tunable range of the P1 oscillation frequency under different injection conditions, the MZM1 bias voltage is swept from 0 to 3 V with a step of 0.1 V for detuning frequencies $f_{i}$ of 9.75, 14.75, 19.75, 24.75, and 29.75 GHz. The measured P1 frequency as a function of MZM1 bias voltage is shown in Figure 2b. Since the injection power decreases with increasing bias voltage, the P1 frequency decreases monotonically as the bias voltage increases. Based on these curves, a pre-distorted driving waveform can be applied to MZM1 to generate a linear frequency-sweeping optical sideband.

According to the measured data, four channels were configured by setting $f_{i}$ to 9.75, 14.75, 24.75, and 29.75 GHz, respectively. The channel with $f_{i}$ = 19.75 GHz is excluded because its measurement range is fully covered by the adjacent channels. The partial overlap between adjacent channels facilitates seamless coverage across the entire 10–48 GHz band, with upper limit constrained by modulator bandwidth rather than the P1 dynamics.

Single-tone frequency measurement is validated across the four channels. A continuous-wave SUT generated by the MSG is applied to MZM2 biased at quadrature transmission point. The period of the drive waveform for MZM1 is fixed at T = 10 μs for all channels. The chirp rate of sweeping sideband is set to 1 GHz/μs for channels 1–3 and increased to 2 GHz/μs for channel 4 to accommodate its broader bandwidth. For channels 1–4, the sweeping sideband reaches terminal frequencies of 19, 26, 34, and 49 GHz, respectively. After reaching the terminal frequency, the sweep is held at this value until the end of the 10-μs period. The SUT frequency is stepped from 10 to 48 GHz with a 1 GHz increment across the four channels, and the system output waveform is recorded by the oscilloscope at a sampling rate of 1 GSa/s.

**Figure 3:**
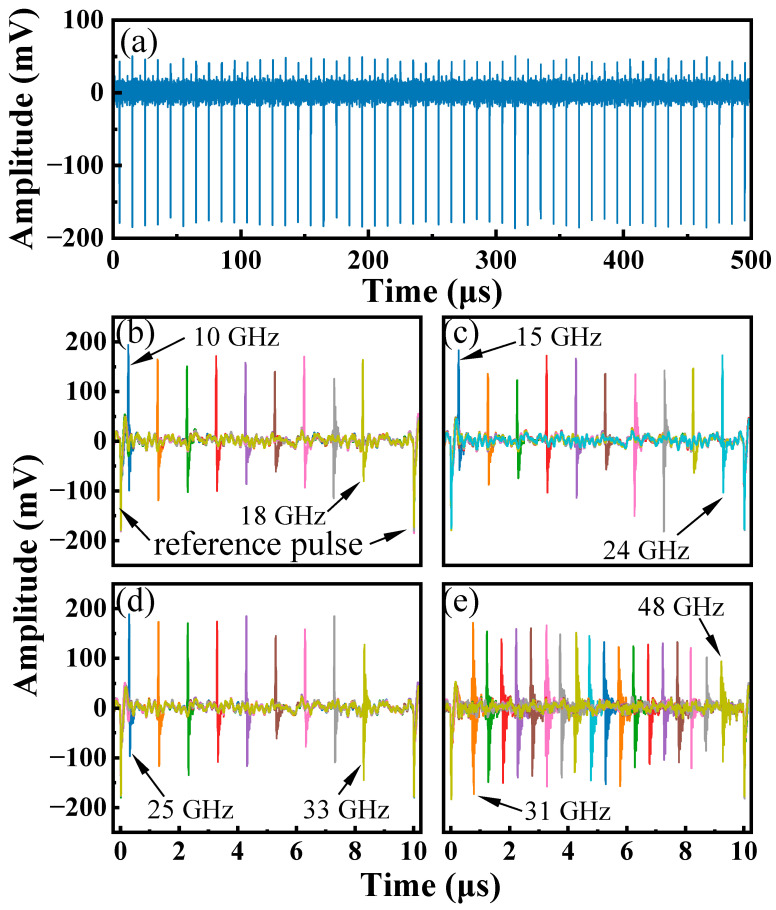
Experimental results of the self-referenced FTTM scheme. (**a**) Recorded waveform over multiple sweep periods without SUT. (**b**–**e**) Single-period waveforms for channels 1–4 with SUT frequency stepped in 1 GHz increments across 10–18, 15–24, 25–33, and 31–48 GHz, respectively. Traces are aligned by the negative reference pulse.

The recorded waveform over multiple sweep periods without SUT input is shown in Figure 3a. The negative pulses appear with a period of 10 μs, consistent with the period of designed driving waveform for MZM1. The amplitude of negative pulses remains stable across multiple sweep periods, indicating that the system can reliably mark the starting point of each sweep period without the need for external synchronization. Representative single-period waveforms for the four channels are presented in Figure 3b–e. For each channel, the SUT frequency was swept across the corresponding measurement range with a 1-GHz step, and a single sweep period was extracted for each frequency point. These traces were aligned by the negative reference pulse and overlaid in each subplot. It should be noted that the similar amplitude distributions of some traces are expected, since the curves in Figure 3b–e are raw time domain waveforms recorded by the oscilloscope. During the measurement, the SUT power, MZM2 bias point, and optical power levels of the selected sidebands were kept similar. Therefore, the beating pulses at different SUT frequencies may exhibit similar amplitudes. In the proposed FTTM scheme, the SUT frequency is determined by the time delay between the negative reference pulse and the positive beating pulse, rather than by the pulse amplitude. In all cases, the negative reference pulse and the positive beating pulse are clearly distinguishable, and the positive pulse position shifts linearly with the SUT frequency, confirming correct self-referenced frequency-to-time mapping. This agrees well with Equation (1), since a higher SUT frequency corresponds to a later overlap between the sweeping sideband and the SUT lower sideband.

The present results also reveal the main limitations of the current implementation. The current upper frequency boundary is set by the bandwidth of MZM2, rather than by the P1 dynamics themselves. The scanning range produced by optical injection is not determined only by the detuning frequency. It also depends on the selected DFB laser and the injection conditions, including the injection strength and its modulation profile. With a more suitable DFB laser and optimized injection parameters, a wider scanning range should be achievable, allowing a single channel to span a broader instantaneous bandwidth and reducing the number of channels required to cover the full range.

**Figure 4:**
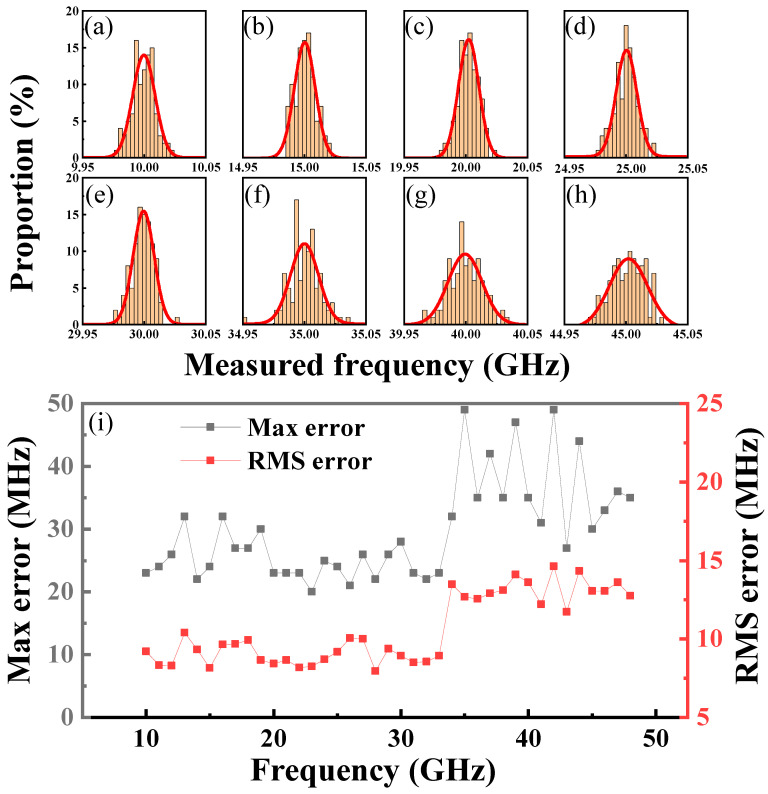
Experimental results for frequency measurement error. (**a**–**h**) Histograms of 100 repeated measurements at representative frequencies for channels 1–4 with normal fits (red curves). (**i**) Maximum absolute error and RMS error across the entire measurement range.

To evaluate measurement repeatability, 100 repeated measurements were performed at each 1-GHz frequency grid across the entire 10–48 GHz range. Figure 4a–h show histograms of the measured frequency distribution at 10, 15, 20, 25, 30, 35, 40, and 45 GHz. The red curves represent Gaussian fits, and all distributions follow a normal distribution regardless of the SUT frequency, indicating that the measurement errors are dominated by random noise rather than systematic bias. The maximum absolute error and root-mean-square (RMS) error for different SUT frequencies are summarized in Figure 4i. The RMS error remains below 15 MHz across the entire measurement range, with a maximum absolute error within 50 MHz.

The dominant error sources can be understood from the frequency-to-time mapping relation in Equation (1). Then, uncertainty in extracting the temporal positions of the negative reference pulse and the positive beating pulse also affects the measured frequency. In this work, the pulse timing is determined from the envelope maximum of the detected pluses, which may slightly deviate from the ideal frequency-matching instant because of phase fluctuation, noise, pulse width, and filtering-induced waveform distortion.

**Figure 5:**
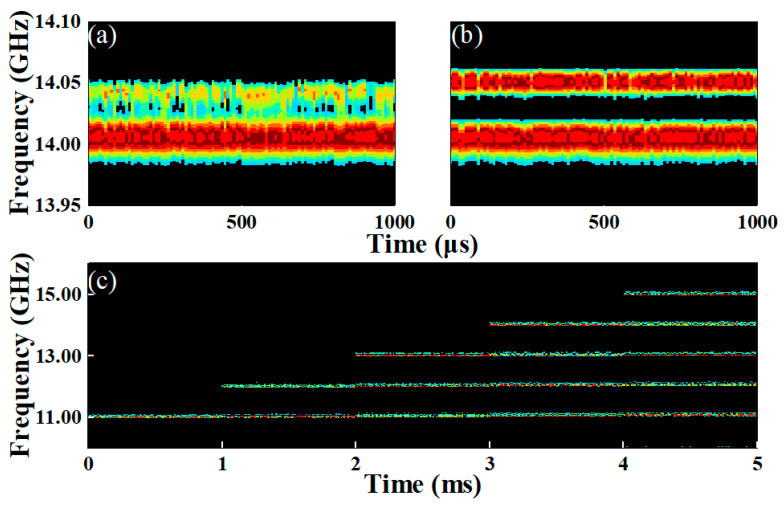
Frequency resolution and multi-tone measurement capability. Time–frequency diagrams of dual-tone signals with frequency separations of (**a**) 40 MHz and (**b**) 50 MHz under a chirp rate of 1 GHz/μs. (**c**) Multi-tone measurement results with the number of simultaneous tones progressively increasing from one to five.

Frequency resolution is tested using dual-tone signals generated by another AWG with a sampling rate of 65 GSa/s. Dual-tone signals with separations of 40 MHz and 50 MHz were applied under the chirp rate of 1 GHz/μs. The output waveforms were aligned by the negative reference pulse, and the instantaneous frequency extracted from each period was mapped using Equation (1) to construct the time–frequency diagram. As shown in Figure 5a, the two frequency components cannot be reliably distinguished when the tone separation is 40 MHz. In contrast, two clearly resolved traces are observed at a separation of 50 MHz, as shown in Figure 5b. The frequency resolution is therefore estimated to be approximately 50 MHz. To evaluate multi-tone measurement capability, signal with progressively increasing number of tones is tested, starting from a single tone and sequentially adding tones up to five simultaneous components. As shown in Figure 5c, all frequency components are correctly identified as the number of tone increases, confirming that the proposed scheme can resolve multiple simultaneous frequencies.

**Figure 6:**
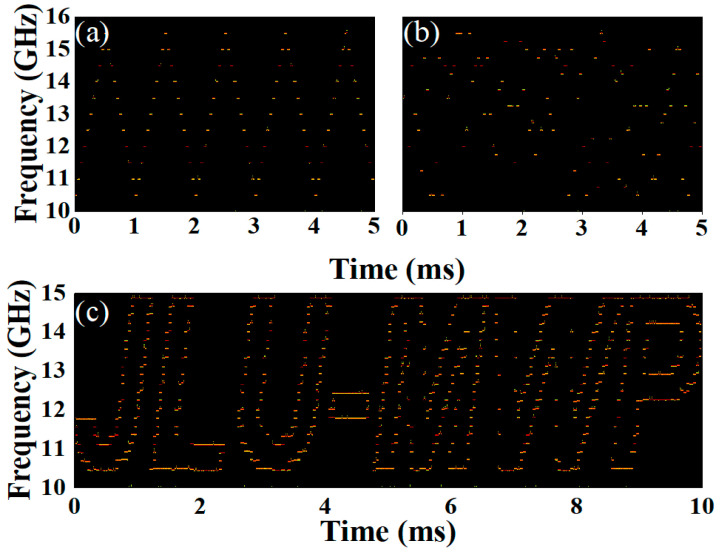
Time–frequency analysis of representative wideband signals. Reconstructed time–frequency diagrams for (**a**) step frequency signal, (**b**) frequency-hopping signal, and (**c**) customized signal forming the pattern “JLU-MWP”.

Time–frequency analysis capability was demonstrated using three representative signals: step frequency signal, frequency-hopping signal, and a customized signal forming the pattern “JLU-MWP” in the time–frequency domain. The step frequency signal consisted of 11 frequency steps with a dwell time of 50 μs per step, while the frequency-hopping signal contained 21 hop frequencies with a hopping rate of 20,000 hops/s. The reconstructed time–frequency diagram shown in Figure 6a–c clearly reveal the frequency evolution of each signal, confirming that the proposed scheme possesses time–frequency analysis capability. It should be noted that the temporal resolution is limited by the sweep period, which is 10 μs in our implementation.

The results show that combining P1-based optical chirp generation with spectral gating can simultaneously alleviate two key constraints in conventional FTTM implementations. On one hand, the frequency sweep is generated optically from the P1 dynamics of the injected DFB laser, so the accessible measurement window is not directly limited by the bandwidth of a high-speed electrical sweep source. On the other hand, the brief interval without injection allows the DFB output to return toward the free-running state. This component is rejected by the OBPF, producing a negative pulse that marks the start of each sweep period. Therefore, no external trigger or dedicated pilot signal is required. In contrast to previous self-referencing methods, which either require a pilot signal [10] or embed a zero-voltage gap into the electrical sweep waveform [12] and therefore still depend on high-speed microwave source, the spectral gating mechanism introduced here derives the temporal marker directly from the optical injection cycle itself. Sweep generation and self-referencing are thus unified in a single all-optical process, simultaneously eliminating the high-speed electrical chirp synthesis requirement and any need for a dedicated trigger signal. As summarized in Table 1, the proposed scheme is compared with representative FTTM-based microwave frequency measurement works. Although the frequency resolution of the proposed scheme is not the highest among the methods listed in Table 1, its advantage lies in the output signal format and the overall functionality. The system produces a low-speed bipolar pulse waveform, where the negative pulse provides an internal temporal reference and the positive pulse indicates the frequency-matched beating event. The reference and signal pulses are therefore distinguished by polarity rather than by amplitude, which eliminates the need for an external trigger, a dedicated pilot tone, or amplitude-based pulse discrimination. In addition, the frequency sweep is generated optically from the P1 dynamics of the injected DFB laser, while the AWG only modulates the injection strength at low speed. This relaxes the bandwidth requirement on the electrical sweep-generation chain and enables a reconfigurable measurement range mainly limited by the DFB laser characteristics and the bandwidth of MZM2. Therefore, the proposed scheme is a balanced solution integrating wide-range measurement, self-referenced operation, low-speed bipolar output, multi-tone measurement, and dynamic time–frequency analysis.

## 4. Conclusions

In summary, a self-referenced microwave frequency measurement scheme with a flexibly reconfigurable range has been demonstrated by combining P1-dynamics-based optical chirp generation with spectral gating in an FTTM architecture. The proposed scheme generates the frequency-sweeping waveform optically. Therefore, the AWG is used only to modulate the injection power at low speed, rather than to directly synthesize a microwave-band chirp. In addition, the temporal reference is generated internally by the spectral-gating-induced negative marker, eliminating the need for external synchronization. The measurement range can be flexibly reconfigured by adjusting the master-laser detuning while the OBPF passband remains fixed; the four-channel implementation demonstrated here covers a total measurement range of 10–48 GHz. Experimental results show a frequency resolution of 50 MHz at a chirp rate of 1 GHz/μs, an RMS error below 15 MHz, and a maximum absolute error within 50 MHz across the full measurement range. The demonstrated capability for multi-tone measurement and time–frequency analysis of complex dynamic signals further confirms the potential of the proposed scheme for wideband microwave signal analysis in radar and electronic warfare applications.

## Figures and Tables

**Table 1:** Comparison with representative photonic microwave frequency measurement and time–frequency analysis schemes.

Ref.	Method	Sweep Period (μs)	Measurement Range (GHz)	Measurement Resolution (MHz)	Measurement Error (MHz)
[10]	AWG + Pilot tone	~1000	1~18	40	10
[11]	AWG + Trigger sync	0.1~1	10–30	30	10~24
[12]	AWG + AM-LFM	4	1~34	60	<20
[13]	P1 + Trigger sync	0.8~1	0~11	200~300	~150
[14]	P1 + Trigger sync	10~50	7.6~47.4	32~90	<108
[15]	P1 + Pulse pair	10	1~39	20	<50
This work	P1 + Spectral gating	10	10~48	50	RMS < 15

## Data Availability

The raw data supporting the conclusions of this article will be made available by the authors on request.
